# Laser Ablating Biomimetic Periodic Array Fish Scale Surface for Drag Reduction

**DOI:** 10.3390/biomimetics9070415

**Published:** 2024-07-07

**Authors:** Dengke Chen, Bowen Zhang, Haifeng Zhang, Zheng Shangguan, Chenggang Sun, Xianxian Cui, Xiaolin Liu, Zehui Zhao, Guang Liu, Huawei Chen

**Affiliations:** 1 College of Transportation, Ludong University, Yantai 264025, China; 2 Shandong Laboratory of Advanced Materials and Green Manufacturing at Yantai, Yantai 264006, China; 3 School of Mechanical Engineering and Automation, Beihang University, Beijing 100191, China; 4 College of Mechanical Engineering, Hebei University of Science & Technology, Shijiazhuang 050091, China; 5 Advanced Innovation Center for Biomedical Engineering, Beihang University, Beijing 100191, China

**Keywords:** laser ablating, fish scale, drag reduction, simulation, vortices

## Abstract

Reducing resistance to surface friction is challenging in the field of engineering. Natural biological systems have evolved unique functional surfaces or special physiological functions to adapt to their complex environments over centuries. Among these biological wonders, fish, one of the oldest in the vertebrate group, have garnered attention due to their exceptional fluid dynamics capabilities. Fish skin has inspired innovation in reducing surface friction due to its unique structures and material properties. Herein, drawing inspiration from the unique properties of fish scales, a periodic array of fish scales was fabricated by laser ablation on a polished aluminum template. The morphology of the biomimetic fish scale surface was characterized using scanning electron microscopy and a white-light interfering profilometer. Drag reduction performance was measured in a closed circulating water tunnel. The maximum drag reduction was 10.26% at a Reynolds number of 39,532, and the drag reduction performance gradually decreased with an increase in the distance between fish scales. The mechanism of the biomimetic drag reduction surface was analyzed using computational fluid dynamics. Streamwise vortices were generated at the valley of the biomimetic fish scale, replacing sliding friction with rolling friction. These results are expected to provide a foundation for in-depth analysis of the hydrodynamic performance of fish and serve as new inspiration for drag reduction and antifouling.

## 1. Introduction

Resistance reduction to surface friction is challenging in a viscous fluid and has attracted considerable attention from researchers in the last few decades [1]. Both active and passive methods have been examined to reduce surface friction [2,3,4]. Because the active drag reduction method requires external energy consumption [5], the passive drag reduction method is trending owing to its lack of external energy consumption over time [6]. Physical creatures have evolved unique surface structural features and physiological functions throughout evolution. Therefore, natural organisms are a source of innovative inspirations for understanding passive drag reduction. Fish are among the most ancient species of the major vertebrate groups; they are a typical representative of excellent fluid dynamics adaptation to a complex environment [7,8,9,10], which can be attributed to both their physiological structure and the unique structure and material properties of the fish skin. Fish skin is a multi-layered system comprising mucus, epidermis, fish scale, and dermis layers from the outer to inner side. Fish scales embedded in a flexible dermis layer serve several functions, such as ensuring resistance to external attacks [11,12] and ion exchange [13]. The drag reduction performance of the fish scale has been examined in recent years. Among aquatic creatures, micro-denticles of sharkskin stand out for their unique structure, conferring exceptional drag reduction and antifouling properties [14,15]. However, not all fish possess these specialized structures. Several fish species exhibit cycloid scales, which have recently garnered attention for their potential role in reducing drag [16,17,18,19]. Certain cycloid-like fish scales have demonstrated drag reduction performance in complex ocean environments.

The drag reduction performance of biomimetic cycloid scale surfaces has been examined in several studies. Wu et al. examined the effect of “water trapping” on drag reduction by crescent microstructures, drawing inspiration from the scales of *Ctenopharyngodon Idella*. The biomimetic surface formed a fluid lubrication film, effectively reducing skin friction drag [20]. Muthuramalingam et al. examined the effect of biomimetic fish scale arrays to reduce drag and reported the formation of high–low speed stripes on the surface, which can delay the boundary transition from laminar to turbulence [17]. Biomimetic fish scales mimicking *Carassius auratus* were fabricated by coating technology for drag reduction [21]. Some studies have employed the laser ablating method as an inexpensive way to fabricate biomimetic functional surfaces. The results suggest that the biomimetic surfaces exhibit different drag reduction performances under various flow conditions [22,23,24]. For instance, Xue et al. developed a fish-skin-inspired Janus hydrogel coating and reported a drag reduction ratio of 38.5% [25]. Chen et al. fabricated biomimetic gradient flexible fish skin with a passive dynamic micro-roughness exhibiting a drag reduction ratio of 13.8% [26]. The nanosecond pulse laser ablation-chemical etching (LACE) process proposed by Liu et al. can be used to prepare patterned superhydrophobic surfaces with microstructures that are orientation-controllable and can be reliably applied to drag reduction and water repellency fields [27]. These biomimetic cycloid fish scale surfaces exhibited a drag reduction effect; however, the influence of fish scale array spacing on drag reduction in relevant papers is rarely reported. Therefore, we fabricated an array of biomimetic fish scales with different spacings and examined the drag reduction performance at different incoming flow velocities.

In this study, five biomimetic periodic array fish scale surfaces with different spacings were fabricated on an aluminum (*Al*) template using the femtosecond laser method. The morphological characteristics of biomimetic fish scale surfaces were characterized using scanning electron microscopy (SEM) and three-dimensional (3D) white-light confocal microscopy devices. Drag reduction performances of the biomimetic fish scale surfaces were examined in a closed circulating water tunnel. A drag reduction of 10.26% was obtained at Reynolds number (*Re*) = 39,532. The computational fluid dynamics (CFDs) method was employed to elucidate the drag reduction mechanism, and the results showed the generation of streamwise vortices on the valley of the fish scale. Thus, the unique hydrodynamic characteristics of the periodic array of the fish scale surface can provide a theoretical reference for understanding the excellent dynamic properties of fish. 

## 2. Materials and Methods

### 2.1. Materials

Albacore tuna was purchased from a seafood market and stored in a freezer before experimentation. A polished *Al* template measuring 50 × 30 × 1 mm^3^ was purchased from the Chaosheng metal market and cleaned using ethanol in an ultrasonicator (CR-020S, Yantai Zhichuang Micro Technic Co., Ltd., Yantai, China). The contact angle (CA) was determined using deionized water at 20 °C, and the droplet volume was 7 μL. Ethanol (analytical grade) and polydimethylsiloxane (PDMS) were obtained from Beijing Chemical Works (Beijing, China) and Sigma-Aldrich Trading Co., Ltd. (Shanghai, China)., respectively. All chemicals were used as received without further processing. 

### 2.2. Biomimetic Prototype and Model Extraction

**Figure 1:**
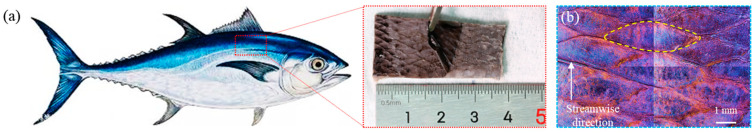
*Albacore tuna* and the arrangement of fish scales. (**a**) *Albacore tuna* and different layers. (**b**) Fish scale arrangement.

In this study, Albacore tuna was the biomimetic prototype. Albacore tuna is known for its high nutritional value and lifelong swimming abilities, with sustained swimming speeds in excess of 1.5 m/s (0.6 body length s^−1^) [28]. The excellent hydrodynamic properties of tuna are attributed to its streamlined body and the unique structure and material properties of its skin. Tuna skin comprises five layers, from outer to inner: mucus, flexible epidermis, fish scale, dermis, and collagen fiber [4]. The hard fish scale layer is covered by a flexible epidermis layer and embedded in the dermis layer, as shown in Figure 1a. The fish scales of the Albacore tuna are not in the micro-dimension (as are the denticles embedded in sharkskin); instead, they are in the millimeter range. The fish scales are arranged in a periodic overlapping pattern, and the posterior region is exposed. This exposed area is shaped like a fan (yellow dotted box), as shown in Figure 1b. The length of the exposed area of the fish scale is longer along the spanwise direction, unlike the streamwise direction, and the line shape of the transition area between fish scales is arched. A biomimetic surface with a periodic array of fish scales was designed based on their arrangement and morphology by studying the fish scale characteristics and the arrangement of tuna skin. 

### 2.3. Sample Preparation

**Figure 2:**
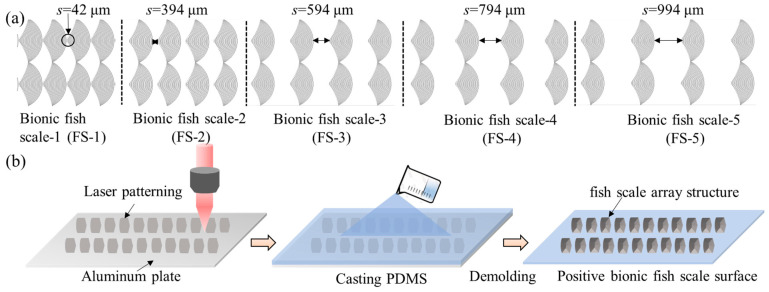
(**a**) Biomimetic fish scale pattern surfaces and (**b**) schematic of the preparation of biomimetic fish scale surfaces.

Firstly, the shape of the biomimetic periodic array fish scale was designed and drawn using CAXA CAD software. The biomimetic periodic array fish scale surface with different spacings comprised multiple curves, and the spacing between adjacent curves was 5 μm. The initial spacing of the fish scale pattern was 42 μm and then gradually increased to 994 μm, as shown in Figure 2a. Secondly, an *Al* plate was cleaned using ethanol in an ultrasonicator for 20 min and dried at 25 °C. A femtosecond laser device (HR-PT-Orien, Yantai, China) was employed to fabricate biomimetic fish scale surfaces following the designed curve. The *Al* plate was placed directly below the laser head, and the distance between the laser source and the sample surface was 152 mm. The laser parameters were set as follows: the pulse duration was set at 1 ps and the processing frequency was 100 kHz. The pulse energy and current were set at 200 μJ and 3 A, respectively, whereas the scanning speed was set at 500 mm/s. Each curve was scanned 30 times. Finally, the *Al* samples fabricated were ultrasonically cleaned and dried in a normal atmospheric environment. Positive biomimetic fish scale surfaces were obtained using a template replication method with PDMS. The ratio of PDMS to curing agent was 10:1. Curing was performed at 80 °C for 2 h, followed by demolding the cured PDMS from the Al template. The preparation process is shown in Figure 2b. 

### 2.4. Sample Characterization

**Figure 3:**
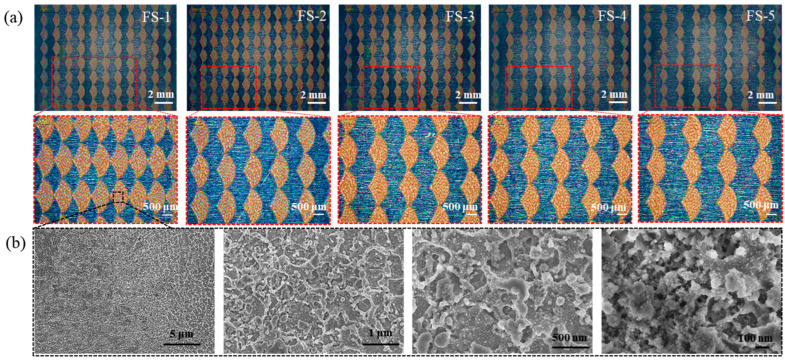
Characteristics of the biomimetic fish scales. (**a**) Optical images of the biomimetic fish scale surface with different spacings. (**b**) SEM images under different magnifications.

A biomimetic fish scale surface was successfully prepared on an *Al* template. The whole size of the fabricated samples measured 50 × 15 mm^2^ (as shown in Supplementary Figure S1). An ultra-depth field microscope (KEYENCE, VHX-970F, Osaka, Japan) was employed to characterize the two-dimensional (2D) morphology of the biomimetic fish scale surface (Figure 3a). The biomimetic fish scales were successfully fabricated and exhibited a periodic array structure on the *Al* plate. To illustrate the microstructure, the positive template of the biomimetic fish scale was processed by gold spraying and characterized using an scanning electron microscope (SEM; Hitachi, SU8010, Osaka, Japan). The results are shown in Figure 3b. The surface roughness of the biomimetic fish scale surface increased, and the microstructures exhibited an irregular porosity feature. The irregular micro-/nanostructures were obtained using a high-energy laser beam.

**Figure 4:**
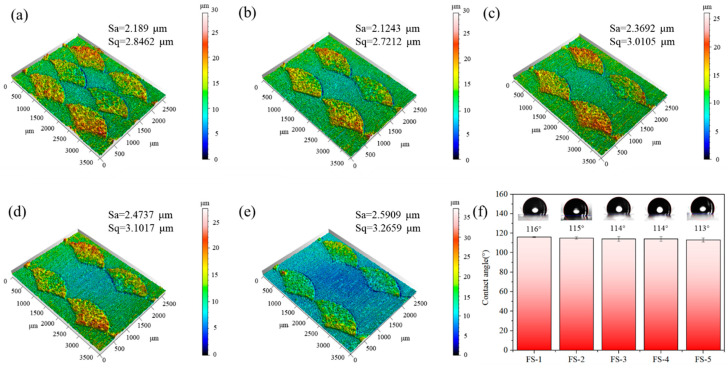
Three-dimensional morphology and contact angle of the biomimetic fish surface. (**a**) FS-1, (**b**) FS-2, (**c**) FS-3, (**d**) FS-4, (**e**) FS-5, and (**f**) contact angle.

The 3D morphology of the biomimetic fish scales was determined using a white-light interfering profilometer (DVM6A, Berlin, Germany). The results are shown in Figure 4a–e. The average height of the fish scale was 25 ± 3 μm and the spacing of these five biomimetic fish scales was different. The surface roughness of the biomimetic fish scales considerably increased after femtosecond laser processing, and the variation in surface roughness is supplied in Supplementary Figure S2. Generally, the surface wettability of a surface with micro-/nanostructures processed by laser often changes [27]. The contact angle (CA) increased with laser processing as micro- or nanostructures exhibited superhydrophobic properties. However, the CAs of biomimetic fish scale surfaces did not exhibit a super-hydrophobic peculiarity and were within 114 ± 2° for all biomimetic surfaces (Figure 4f). Our results suggest that the arrangement of the fish scales is the only factor affecting drag reduction.

### 2.5. Drag Measurement

**Figure 5:**
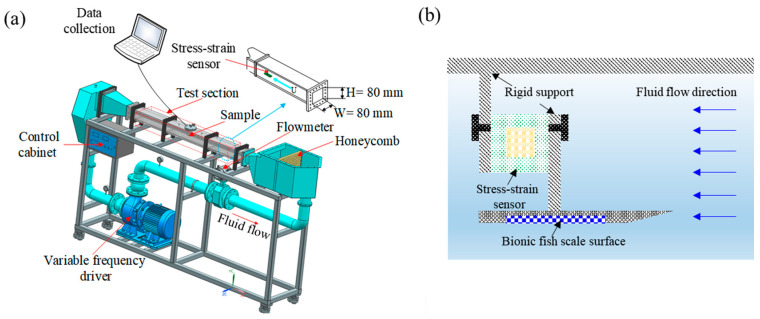
Schematic of the closed circulating water tunnel. (**a**) 3D diagram of the water tunnel. (**b**) Schematic of the biomimetic surface and stress–strain sensor installation.

A closed circulating water tunnel was employed to measure the total drag force of smooth and biomimetic fish scale surfaces. Figure 5 shows the schematic of the closed circulating water tunnel and test section. Fabricated PDMS samples were placed in a groove of the mold, and the direction of the biomimetic fish scale was parallel to that of the flow. The fabricated PDMS smooth surface had no biomimetic fish scale. The mold was rigidly connected to a stress–strain sensor, a unidirectional force device having an accuracy of 0.01 mN and a measuring range of 0–20 N, to measure the total drag force of the biomimetic fish scale surface and placed in the middle of the test section. The stress–strain sensor was a one-way force device, which means that the deformation only can be generated in the fluid flow direction. The accuracy of the stress–strain sensor was evaluated according to the voltage signal generated by weights of 0, 50, 100, and 200 g before the test. The test section comprised transparent acrylic and the total length was 1500 mm. The width (*w*) and height (*h*) of the test sections were 80 and 80 mm, respectively. The total drag force of biomimetic fish scale surfaces was measured at different incoming flow velocities (0.5, 1, 1.5, and 2 m/s). Tap water was the working media and the velocity of water was controlled using a variable frequency driver. The water inlet remained open and excess tap water was discharged through the overflow port to limit the operating temperature of the tap water. The temperature of the water was monitored using a thermometer and the tap water temperature was maintained at 25 ± 2 °C, to exclude the effect of water temperature on the experimental results. Multiple honeycomb turbulators were set at the inlet chest to stabilize the flow field and reduce the turbulence intensity. The Reynolds (*Re*) number corresponding to the different velocities was calculated using the following formula:

(1) Re=ρUD/u

where ρ, *U*, *D*, and *u* are the water density, flow velocity, hydraulic diameter, and the dynamic viscosity, respectively. The hydraulic diameter was determined as follows: *D* = 2*wh*/(*w* + *h*). The calculated *Re* numbers were 39,532, 79,065, 118,598, and 158,130, indicating fully turbulent flow in the test section (*Re*_critical_ = 2300). 

## 3. Drag Reduction

**Figure 6:**
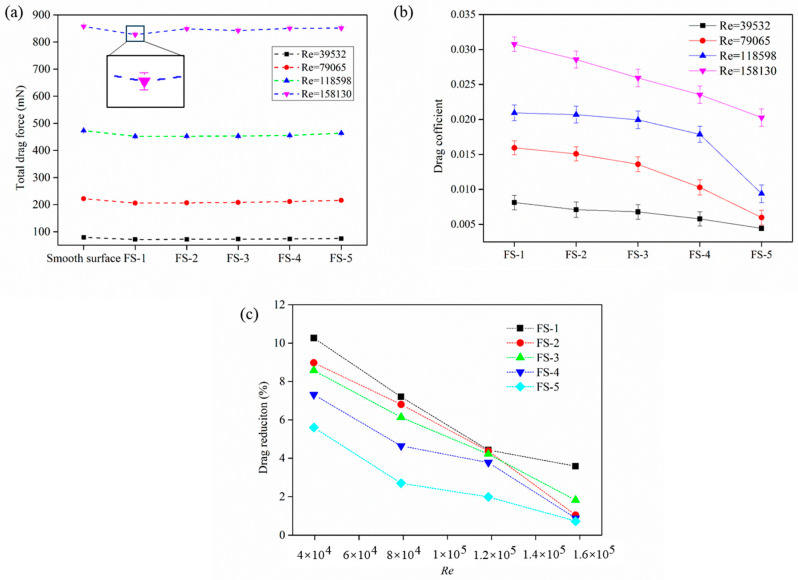
Results of test. (**a**) Total drag force for biomimetic fish scale at different *Re* numbers. (**b**) Drag coefficient of the biomimetic fish scale surface. (**c**) Drag reduction rate.

The total drag force of the biomimetic fish scale surfaces was measured and all biomimetic samples were tested thrice. The average value was considered as the final value. The total drag force of the smooth surface at different *Re* values was measured and considered the contrast value (Figure S3). The drag reduction rate was calculated as per Equation (2):

(2) $$\mathrm{Drag}\mathrm{reduction}\;(\mathrm{DR}\%)\;=\frac{F_{\mathrm{smooth}}-F_{\mathrm{biomimetic}}}{F_{\mathrm{smooth}}}\times 100\%$$

where *F_smooth_* and *F_biomimetic_* are the total drag force of the smooth surface and biomimetic fish scale surface, respectively. Figure 6a shows the total drag force of the smooth and biomimetic surfaces. The resulting error bar is extremely small compared to the ordinate value and cannot be obviously seen in Figure 6a. The results showed that the total drag force significantly increased with Re for all surfaces, with the total drag force of the smooth surface being the greatest and that of the FS-1 surface being the least. Furthermore, the total drag force gradually increased as the fish scale spacing increased, approaching the drag force value of the smooth surface. The drag reduction rate of the biomimetic fish scale surface was obtained using Formula (2) (Figure 6b). The best drag reduction performance of the biomimetic fish scale surface was FS-1 at *Re* = 39,532, and the drag reduction rate gradually decreased with an increase in spacing under the same *Re* numbers. The maximum value *DR* of FS-1 was 10.26% at *Re* = 39,532. 

## 4. Mechanism 

### 4.1. Simulation Model

**Figure 7:**
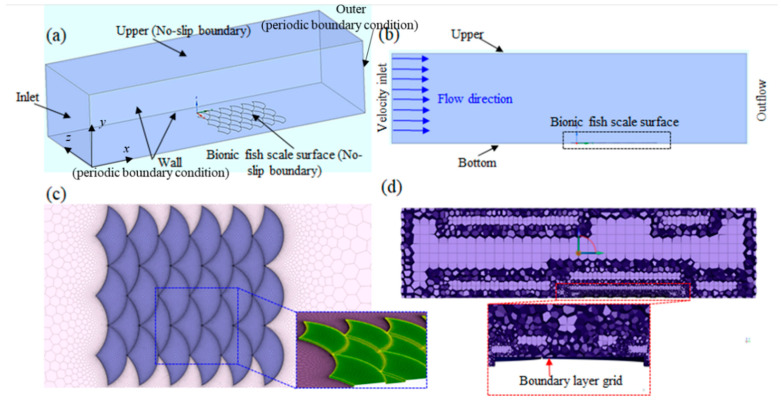
Simulation model and boundary conditions. (**a**) Boundary conditions for the numerical simulation of the biomimetic fish scale surface. (**b**) Computational domain for the numerical simulation of the biomimetic fish scale surface. (**c**) Polyhexcore grid of the biomimetic fish scale domain. (**d**) Fluid domain grid with a boundary layer.

The CFD method was employed to calculate the fluid flow of the biomimetic fish scales near the wall to elucidate the mechanism of drag reduction in the biomimetic fish scale surface in turbulent flow. First, the numerical simulation models of the biomimetic fish scale and fluid domain were established (Figure 7a,b). Setting reasonable and accurate boundary conditions and calculation methods was critical to the calculation. The boundary conditions were defined such that the inlet used was the velocity inlet, the outlet used was the outflow boundary condition, the upper and biomimetic fish scale surfaces were in the no-slip boundary condition, and the wall and outlet used were in the periodic boundary conditions (Figure 7a). The fluid domain is shown Figure 7b. Reasonable fluid domain size was important for calculations. Therefore, the size of the fluid domain was *L_x_ × L_y_ × L_z_* = 16*H*′ × 4*H*′ × 2*H*′, where *H*′ represents the half-width of the fluid domain channel. The height of the fluid domain must exceed ten times *h* of the biomimetic fish scale to prevent interference with the flow field near the fish scale wall. A transition zone (8*H*′) was established between the biomimetic fish scale surface and the inlet to ensure a smooth transition in fluid flow. The working fluid was water flowing horizontally along the *x*-direction (streamwise direction). The inlet velocity ranged from 0 to 2 m/s. Fluent meshing (2021 R1) was employed to generate a polyhexcore grid (Figure 7c). A non-uniform boundary layer of gird was applied on the biomimetic fish scale surface in the wall-normal direction to obtain the precision flow field near the biomimetic fish scale wall. The first layer grid distance was obtained using a *y*+ calculator and was 2.3 × 10^−5^ m. The grid spacing increased from the walls by an aspect ratio of 1.1 with 10 boundary layers. The schematic of the biomimetic fish scale surface with a non-uniform boundary layer is illustrated in Figure 7d. 

**Figure 8:**
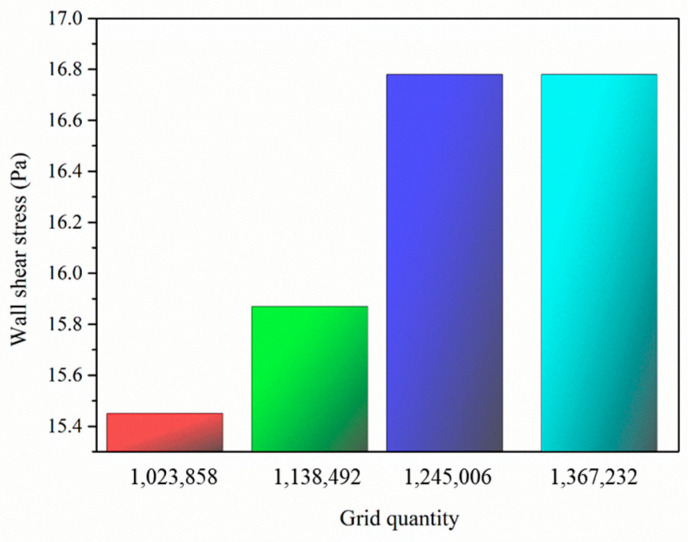
Independence validation of the grid density.

The Large Eddy Simulation (LES) turbulent model and wall-adapting local eddy-viscosity subgrid-scale model were used to analyze the flow field characteristic [29,30,31]. Despite the complexity of the turbulent flow in the test section, it satisfies the basic governing equations of fluid mechanics. The incompressible Navier–Stokes equation can be expressed as follows:

(3) $$\frac{\vartheta u_{i}}{\vartheta x_{i}}=0,$$

(4) $$\frac{\vartheta u_{i}}{\vartheta t}+\frac{\vartheta u_{i}u_{j}}{\vartheta x_{j}}=-\frac{1}{\rho }\frac{\vartheta p}{\vartheta x_{i}}+v\frac{\vartheta^{2}u_{i}}{\vartheta x_{i}\vartheta x_{j}}{+\;f}_{i}$$

where $u_{i}$ is the velocity components of a fluid in different directions; $x_{i}$ is the spatial coordinate in different directions; *t* is the time; ρ is the fluid density; *p* is the pressure of the fluid; *v* is the viscosity of the fluid; and *f_i_* represents the volume force per unit mass acting on a fluid element in different directions. Pressure−velocity coupling was modeled by the pressure implicit in the splitting of the operator’s algorithm. The momentum equation was discretized using bounded central differencing, and the time term was solved by a second-order implicit method for higher accuracy. Appropriate boundary conditions and grids were established to ensure convergent and consistent results throughout the simulation. Therefore, the independence validation of grid density on the area–weight wall shear stress was analyzed, as shown in Figure 8. The shear stress of the biomimetic periodic array fish scale surface stabilized when the grid number reached 1,245,006 at a velocity of 2.5 m/s. Thus, the number of grid elements should not be less than 1,245,006 in the simulation. When the residual values of continuity, *x*-velocity, *y*-velocity, and *z*-velocity were less than 1 × 10^−3^, the calculation result was considered convergent. 

### 4.2. Flow Characteristics

**Figure 9:**
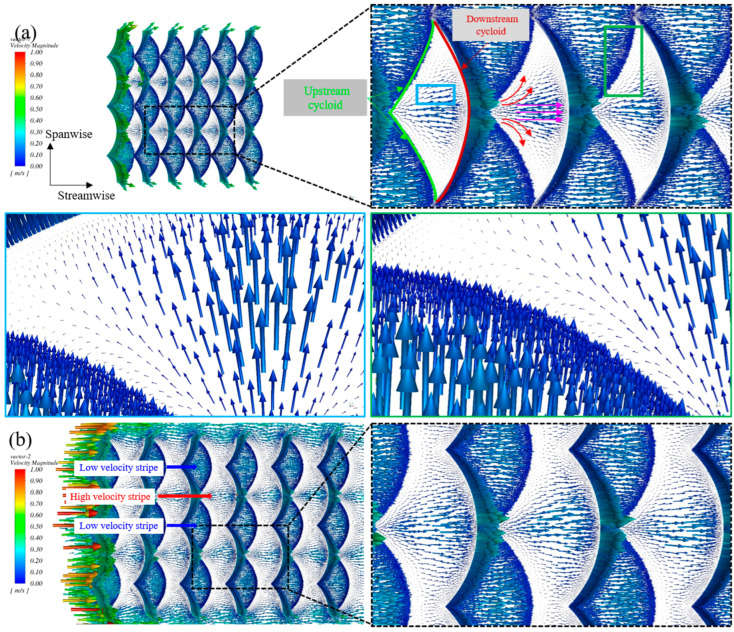
The velocity magnitude vector of the biomimetic fish scale. (**a**) Velocity magnitude vector of FS-1 and a fluid flow magnified drawing. (**b**) Velocity magnitude vector of FS-2 and a magnified drawing.

The flow field characteristics near the wall were important to reveal the drag reduction mechanism of the biomimetic fish scale. Therefore, simulation results were processed using Fluent post-processing software, and the velocity vector of the biomimetic fish scale surface was obtained. The velocity magnitude vectors of FS-1 and FS-2 were selected and processed to explain why the drag reduction performance of FS-1 was the best, and the drag reduction effect gradually decreased with an increase in the spacing of the fish scale (Figure 9). The incoming flow near the biomimetic fish scale wall was divided into two parts when the spacing of the fish scale was the least (FS-1): one part directly flowed along the streamwise direction (pink arrows), and this fluid moved along the streamwise direction to the midpoint of the downstream cycloid of the fish scale (Enlarged blue box in Figure 9a). When this part of the fluid moved to the trough of the downstream cycloid, it was lifted due to the effect of the fish scale structure, which reduced the normal velocity gradient, thus decreasing the wall friction resistance. The other part flows along the upstream cycloid of the fish scale (red arrows) (enlarged green box in Figure 9a). This part of the flowing fluid was divided into two parts. The first part of the fluid flow moved to the upstream cycloid and merged with the incoming flow. Another part of the fluid flow also participated in the flow along the upstream cycloid, and vortices formed on the valley of the biomimetic fish scale, leading to sliding friction being replaced with rolling friction. In addition, the high–low velocity stripes were formed on the biomimetic fish scale surface due to the fluid dividing and merging with the incoming flow (Figure 9b). The formed velocity stripes were directly associated with the path of the fluid flow. The first part (pink arrows) flowed directly along the streamwise direction, and the velocity magnitude vector was significantly larger than those in the other positions. The second part (red arrows) participated in the formation of the vortices and merged with the incoming flow. Therefore, this special flow weakened the velocity magnitude along the streamwise direction, as indicated by the velocity magnitude vector of this part of the merged fluid being smaller than that of the first part. This is the direct reason for the function of the delay transition of high–low velocity stripes and the high–low velocity stripes, which further reduced the drag [17]. However, the number of vortices was not related to the increase in the spacing of the fish scales, and they mostly flowed along the streamwise direction, as shown in Figure 9b. 

**Figure 10:**
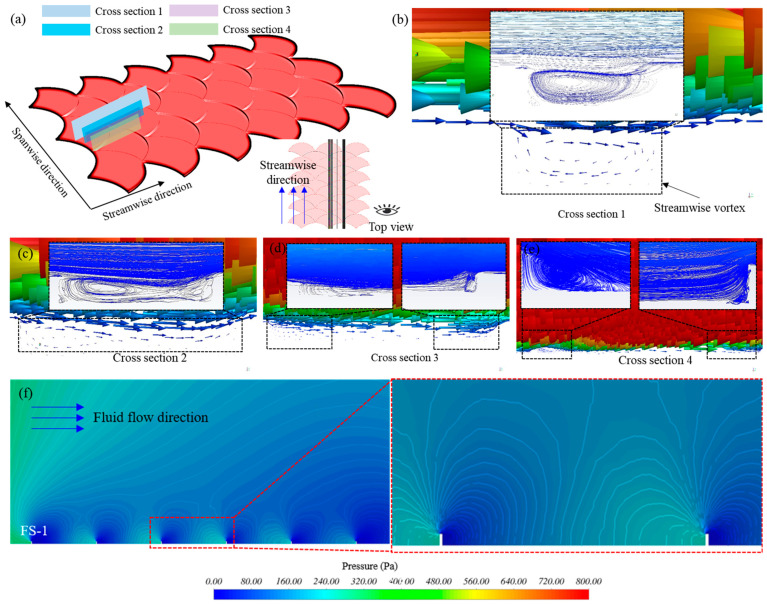
Velocity magnitude vector and pathlines for the biomimetic fish scale surface. (**a**) Position diagram of the cross section on the biomimetic fish scale. (**b**) Streamwise vortices at section 1. (**c**–**e**) Vortices at cross sections 2, 3, and 4, respectively. (**f**) The distribution of the FS-1 surface.

Remarkably, when the incoming fluid flowed along the upstream cycloid of FS-1, the flowing fluid moved to the tip of the fish scale. Owing to the hindrance of the fluid by the fish scale, some fluid mixed with the incoming flow. However, this mixed fluid did not form a high-velocity stripe. Nevertheless, the other part generated streamwise vortices under the influence of the unique biomimetic fish scale. Four cross sections along streamwise direction were established on the biomimetic fish scale surface to reveal the characteristics of the flow field near the fish scale wall (Figure 10a). Cross section 1 as the narrowest tip of a fish scale, cross sections 2 and 3 as the middle position of the cycloid, and cross section 4 at the middle position of the adjacent two fish scales. As described above, with the fluid flow moving along the cycloid to the tip-region-adjacent fish scales, a large streamwise vortex was generated (Figure 10b, the enlarged box was the velocity pathline). Streamwise vortices were generated on the valley of the fish scale along the cycloid, which can be observed in cross sections 2 and 3, as shown in Figure 10c,d. Interestingly, a couple of streamwise vortices formed at the valley of the adjacent fish scale along the streamwise direction, as shown in Figure 10e. In contrast, the streamwise vortices were not generated on the adjacent fish scale of FS-2 in cross section 1, which was the most mobile fluid of part two, and directly merged with the incoming flow. However, streamwise vortices were generated on the valley of the biomimetic fish scale, as shown in Supplementary Figure S4a. In the other two sections, the flow field characteristics were roughly similar to FS-1, as shown in Supplementary Figure S4b,c. These streamwise vortices acted as rolling bearings to reduce the drag friction between the biomimetic fish scale surface and water [32,33,34]. The number of streamwise vortices of the FS-1 was highest in all biomimetic fish scale surfaces; therefore, FS-1 showed the best drag reduction performance. In addition, the biomimetic fish scale unit cells changed the wall pressure distribution, and a peak pressure point was formed in front of the biomimetic fish scale unit cells (upstream side); the difference in the pressure distribution in the space between the fish scales became more tangible [32,34], as shown in Figure 10f.

## 5. Conclusions

In summary, the unique fish scale structure of *Albacore tuna* was used as the biomimetic prototype and a biomimetic periodic array fish scale surface was designed in this study. The biomimetic fish scale surface was fabricated using a femtosecond laser device. The features of the morphology of the biomimetic fish scale were characterized using SEM and a white-light interfering profilometer. The characterization results showed that the height of the biomimetic fish scale prepared at the same level and the surface roughness considerably increased. The drag reduction performance of the biomimetic fish scale surfaces was measured in a circulating water tunnel. The FS-1 surface showed the maximum drag reduction rate of 10.26% at *Re* = 39,532. The mechanism of action of the biomimetic fish scale was revealed using the CFD method. Rolling bearings replaced the sliding friction, which led to the biomimetic fish scale exerting a drag reduction effect. These results can serve as a foundation for an in-depth analysis of the hydrodynamic performance of fish and a novel inspiration for drag reduction and antifouling.

## Data Availability

The data that support the findings of this study are available from the corresponding author upon reasonable request.

## Supplementary Materials

The following supporting information can be downloaded at: https://www.mdpi.com/article/10.3390/biomimetics9070415/s1, Figure S1: Bionic fish scale surface fabricated on Al template; Figure S2: Diagram of section position and the height variation trend. Figure S3: Total drag force of smooth surface at different velocities; Figure S4: Streamwise vortices of FS-2 surface at different cross sections.
