# Supplementary material for: Laser Ablating Biomimetic Periodic Array Fish Scale Surface for Drag Reduction
Source: Biomimetics (Basel). 2024 Jul 7;9(7):415. doi: 10.3390/biomimetics9070415 (PMC11274741; doi:10.3390/biomimetics9070415)
Supplement: Supplementary file 1 [file biomimetics-09-00415-s001.zip › biomimetics-3065603-supplementary.pdf]

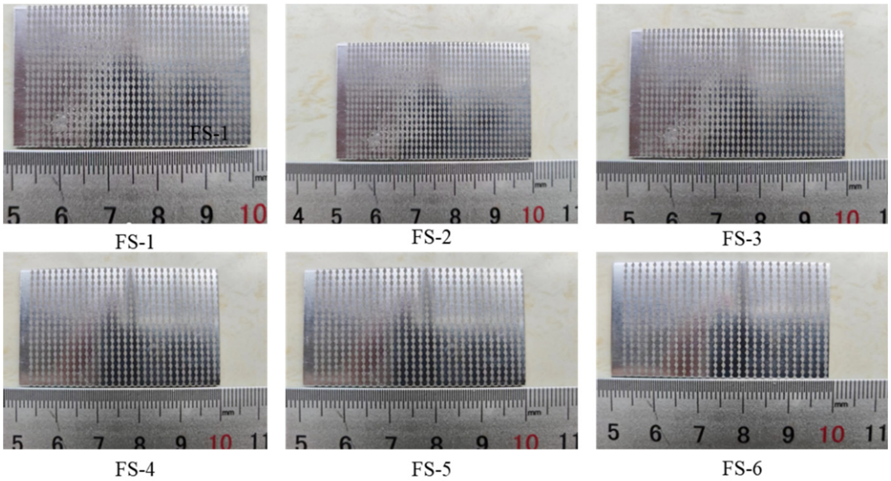

Figure. S1 Bionic fish scale surface fabricated on Al template

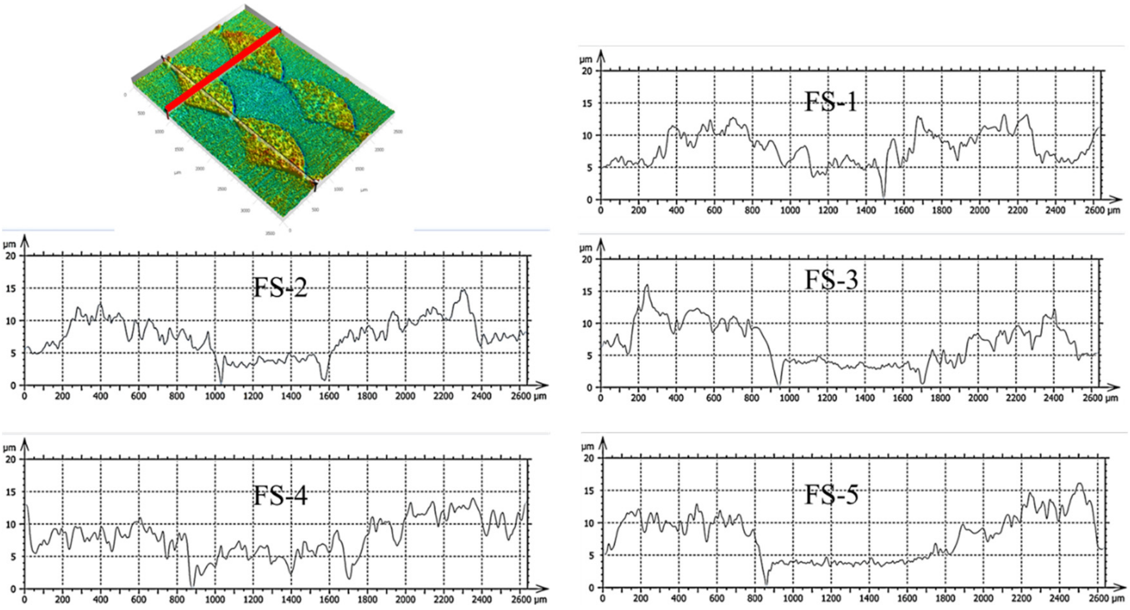

Figure. S2 Diagram of section position and the height variation trend.

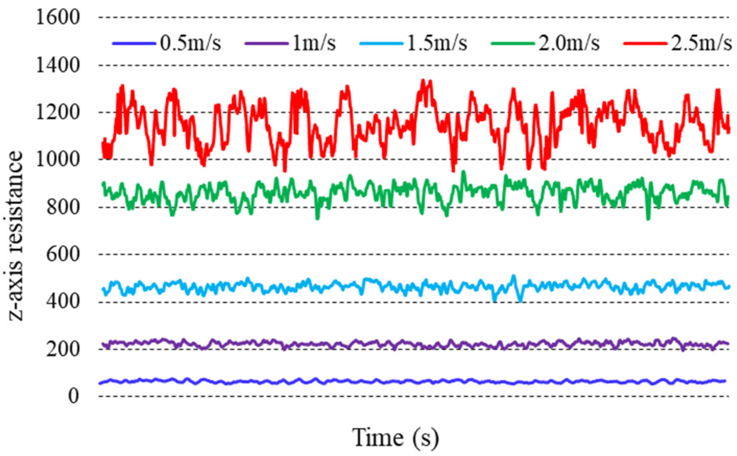

Figure. S3 Total drag force of smooth surface at different velocities

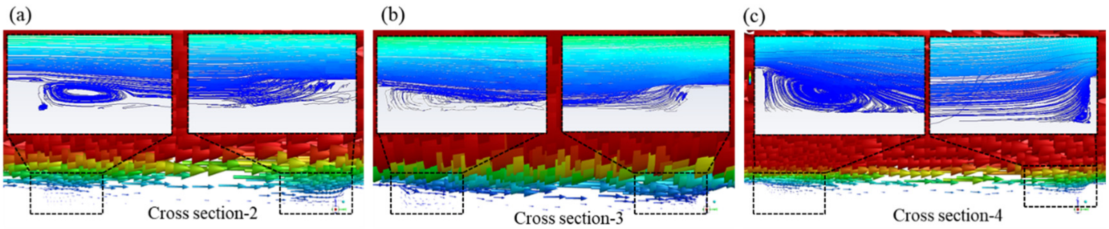

Figure S4 (a)~(c) Streamwise vortices of FS-2 surface at different cross sections.
